# Enhancing the use of evidence-based decision-making processes to establish immunization policies and programmes at national level: what should be the roe of Pasteur Institutes?

**Published:** 2011-01-10

**Authors:** Kamel Senouci, Mireille Dosso, Nguyen Tran Hien, Nyambat Batmunkh, Papa Coumba Faye, Julia Blau, Don Douglas, Alfred Da Silva, Bradford Gessner

**Affiliations:** 1 Agence de Medecine Preventive, s/c Institut Pasteur, 75015 Paris, France; 2 Institut Pasteur de Côte d’Ivoire, 01 BP 490, Abidjan 01, Ivory Coast; 3 National Institute of Health and Epidemiology, Hanoi, Vietnam; 4 International Vaccine Institute, Gwanak-gu, Seoul, Korea 151-919

## 

In both developed and developing countries, the need for evidence-based decision-making in immunization programs has become crucial in light of multiple health priorities, limited human resources and logistical capacities, as well as the high cost of vaccines relative to limited public funds that are available. An important step that countries can take to encourage well-informed decision-making is to establish a group of national experts to advise the policy makers at the Ministry of Health and program managers for making decisions, including choices of new vaccines and needed adjustments to existing programs and schedules.

So far, many countries have already constituted such National Immunization Technical Advisory Groups (NITAGs) while other countries are currently working towards their establishment with the support of technical partners such as WHO and AMP (SIVAC Initiative). Based on WHO recommendations, the composition of the advisory group should include two categories of members: core and non-core members. All core members should be independent and credible experts who serve in their own capacity and who do not represent the interests of a particular group. Core members only, should participate in advising and deciding on the final set of recommendations. Non-core members hold key positions with important government entities they represent (e.g. Outbreaks Surveillance Department, National Drugs Regulatory Authorities, National Public Health Laboratory etc.) and represent various important professional societies, and key technical partners (e.g. WHO and UNICEF).

It is recommended that the committee be multidisciplinary and represent a broad range of expertise from the following disciplines: clinical medicine (pediatrics medicine, adult medicine, geriatrics), epidemiologists, infectious diseases specialists, microbiologists, public health, immunology, vaccinology. Pasteur Institutes have a crucial role to play in the NITAGs’ activities. They can either be sought to participate as non-core member or to send experts as core members.

As non-core member, Pasteur Institutes provide data regarding burden of disease related to their laboratory surveillance activities. They will also provide specific information regarding immunology and safety issues related to vaccines. They can also provide information regarding emerging diseases and therefore influence the national research agenda by having the NITAG issuing specific recommendations. As core members, the experts from Pasteur Institutes should refrain from promoting the policies and views and products of the organization but this leads to an indirect recognition of the expertise of the institution.

As shown in other countries, it also may conduct to NITAG formal request regarding surveys and studies related to vaccines to the institution. Among other countries, the SIVAC Initiative is currently supporting Cote d’Ivoire and Viet Nam in establishing and strengthening NITAGs. In both countries, Pasteur Institutes already participate actively to the process and should be able to share their experience with other institutes of the Pasteur network.

